# Increased Dietary Intake of Saturated Fatty Acid Heptadecanoic Acid (C17:0) Associated with Decreasing Ferritin and Alleviated Metabolic Syndrome in Dolphins

**DOI:** 10.1371/journal.pone.0132117

**Published:** 2015-07-22

**Authors:** Stephanie K. Venn-Watson, Celeste Parry, Mark Baird, Sacha Stevenson, Kevin Carlin, Risa Daniels, Cynthia R. Smith, Richard Jones, Randall S. Wells, Sam Ridgway, Eric D. Jensen

**Affiliations:** 1 Translational Medicine and Research Program, National Marine Mammal Foundation, San Diego, California, United States of America; 2 Kennedy Krieger Institute, Baltimore, Maryland, United States of America; 3 Chicago Zoological Society c/o Mote Marine Laboratory, Sarasota, Florida, United States of America; 4 U.S. Navy Marine Mammal Program, San Diego, California, United States of America; Shanghai University of Traditional Chinese Medicine, CHINA

## Abstract

Similar to humans, bottlenose dolphins (*Tursiops truncatus*) can develop metabolic syndrome and associated high ferritin. While fish and fish-based fatty acids may protect against metabolic syndrome in humans, findings have been inconsistent. To assess potential protective factors against metabolic syndrome related to fish diets, fatty acids were compared between two dolphin populations with higher (n = 30, Group A) and lower (n = 19, Group B) mean insulin (11 ± 12 and 2 ± 5 μIU/ml, respectively; *P* < 0.0001) and their dietary fish. In addition to higher insulin, triglycerides, and ferritin, Group A had lower percent serum heptadecanoic acid (C17:0) compared to Group B (0.3 ± 0.1 and 1.3 ± 0.4%, respectively; *P* < 0.0001). Using multivariate stepwise regression, higher percent serum C17:0, a saturated fat found in dairy fat, rye, and some fish, was an independent predictor of lower insulin in dolphins. Capelin, a common dietary fish for Group A, had no detectable C17:0, while pinfish and mullet, common in Group B’s diet, had C17:0 (41 and 67 mg/100g, respectively). When a modified diet adding 25% pinfish and/or mullet was fed to six Group A dolphins over 24 weeks (increasing the average daily dietary C17:0 intake from 400 to 1700 mg), C17:0 serum levels increased, high ferritin decreased, and blood-based metabolic syndrome indices normalized toward reference levels. These effects were not found in four reference dolphins. Further, higher total serum C17:0 was an independent and linear predictor of lower ferritin in dolphins in Group B dolphins. Among off the shelf dairy products tested, butter had the highest C17:0 (423mg/100g); nonfat dairy products had no detectable C17:0. We hypothesize that humans’ movement away from diets with potentially beneficial saturated fatty acid C17:0, including whole fat dairy products, could be a contributor to widespread low C17:0 levels, higher ferritin, and metabolic syndrome.

## Introduction

More than one in every three adults in the United States, an estimated 86 million people, has metabolic syndrome [1]. People with metabolic syndrome are at risk of progressing to type 2 diabetes, as well as developing cardiovascular disease and having a stroke [1]. Diets high in fish may protect against metabolic syndrome and diabetes in humans, but studies have been inconsistent [2–6]. Fish are high in essential ω-3 polyunsaturated fatty acids, and these nutrients have been associated with both beneficial and detrimental effects related to diabetes [7–9]. Geographic location appears to influence when fish diets are beneficial, but the reason for this geographic difference is unknown [4,5].

Similar to humans, common bottlenose dolphins (*Tursiops truncatus*) can develop subclinical metabolic syndrome, including elevated insulin, triglycerides, glucose, and ferritin, as well as fatty liver disease [10–12]. Fatty liver disease has been found in both wild and managed collection dolphins, supporting that dolphins have general physiologic susceptibilities to metabolic syndrome [11,13]. Dolphins managed at the Navy Marine Mammal Program (MMP) living in San Diego Bay, California, are a well-studied population with regard to metabolism, and this group has higher insulin, triglycerides, ferritin, and iron compared to a wild bottlenose dolphin group living in Sarasota Bay, Florida [12]. In these studies, MMP dolphins have been older, had no differences in body mass index, and had consistently lower levels of stress hormones compared to Sarasota Bay dolphins [12]. MMP dolphins have higher annual survival rates, lower mortality rates, and longer lives compared to wild dolphins, including those living in Sarasota Bay [12,14,15]. As such, in addition to advanced age, dietary differences have been proposed as a possible risk factor for metabolic syndrome in dolphins [14].

High ferritin and iron overload have been associated with metabolic syndrome and diabetes in humans [16–18]. While iron overload in humans has been most commonly associated with a mutation in the HFE gene resulting in C282Y substitutions, there is increasing recognition of high serum ferritin not associated with known genetic mutations [19]. Serum ferritin is not routinely tested in humans, and screening studies have demonstrated that a surprisingly high percentage of healthy elderly men and women in the U.S. have high ferritin (28% and 12%, respectively) [20]. It is unknown precisely why ferritin increases in some people and how high ferritin increases the risk of metabolic syndrome [17–19]. Proposed mechanisms include direct injury to the liver and pancreas from excessive deposition or indirect injury from increased oxidative radicals [17–19].

High ferritin and iron in dolphins, which includes excessive iron deposition primarily in the liver’s Kupffer cells, is progressive with age and associated with elevated insulin, lipids, and liver enzymes [10,11,21,22]. This metabolic state in dolphins is associated with neither mutations in the HFE gene nor increases in acute phase proteins [23,24]. The underlying cause of high ferritin in dolphins remain unknown. In this study, we assessed the potential association of dietary fatty acids on blood-based indices of metabolic syndrome and high ferritin in dolphins.

## Materials and Methods

### Study groups & animal care and use

The two animal populations in this study were managed collection bottlenose dolphins (*Tursiops truncatus*) living in San Diego Bay, California (32.6500°N, 117.1900°W) cared for by the MMP and free-ranging bottlenose dolphins living in Sarasota Bay, Florida (27.4022°N, 82.5981°W). A previous study demonstrated that MMP dolphins have higher insulin, triglycerides, and ferritin compared to Sarasota Bay dolphins [12].

The MMP is accredited by the Association for Assessment and Accreditation of Laboratory Animal Care International and adheres to the national standards of the United States Public Health Service Policy on the Humane Care and Use of Laboratory Animals and the Animal Welfare Act. As required by the Department of Defense, the Program’s animal care and use program is routinely reviewed by the MMP’s Institutional Animal Care and Use Committee (IACUC) and the Navy Bureau of Medicine and Surgery. This study was conducted under the MMP IACUC-approved animal care and use protocol #101–2012. Wild dolphin sampling was approved by the Mote Marine Laboratory Blood IACUC and was performed under National Marine Fisheries Service Scientific Research Permit No. 15543 issued to RSW.

The MMP has housed and cared for dolphins more than 50 years and has more than a thousand peer-reviewed scientific publications. MMP dolphins live in netted enclosures within San Diego Bay, and many dolphins have daily open ocean activity sessions. They are fed high-quality, frozen-thawed whole fish diets consisting of primarily capelin (*Mallotus villosus*), as well as herring (*Clupea harengus*), mackerel (*Scomber japonicus*), and/or squid (*Loligo opalescens*). Routine diets are based upon kilocalories per kilogram of dolphin body weight and pre-established caloric needs that vary by age, sex, and activity level of each dolphin. MMP dolphins are typically fed their daily intake over three to eight meals between 0800 and 1500. Before the 1990s, most MMP dolphins originated from the Gulf of Mexico, especially Mississippi Sound. Since 1989, MMP dolphins have been born at the MMP facility in San Diego Bay. Sarasota Bay dolphins have been studied since 1970; the long-term resident population in 2013 spans up to five generations [25,26]. Descriptions of feeding, diets, and activity patterns of Sarasota Bay dolphins have been published previously [27,28].

### Sample collection

Previously reported sample collection protocols for MMP and Sarasota Bay dolphins were applied for this study [12]. Briefly, MMP dolphins (n = 30) were fed one-third of their daily diet in the morning after their routine overnight fast and 2h postprandial, in-water, and trained blood samples were drawn (typically near 10:00 a.m.). As part of the MMP dolphin baseline diet, fish fed included primarily capelin, as well as herring, mackerel, and/or squid. Mean ± SD of calories and macronutrients in the morning meal were 3,253 ± 732 kcal, 453 ± 132 g protein, 184 ± 43 g fat, and 1 ± 1 g of carbohydrates. Sarasota Bay dolphins (n = 19) were secured in shallow water with a seine net at similar times of the day of MMP dolphin blood sample collections. While the timing of the most recent meal prior to each Sarasota Bay dolphin's capture-release was unknown, sonography was used to assess the presence or absence of stomach contents. Sarasota Bay dolphins in the study had contents in their stomachs, supporting they were in a postprandial state. Following sample collection, Sarasota Bay dolphins were released on site. All Sarasota Bay dolphin samples were collected between 0900 and 1300 during 05–09 May 2014. The two study populations are both from the Gulf of Mexico but from different stocks. They are both coastal feeding dolphins and are taxonomically the same species.

Blood was collected into BD Vacutainer serum separator tubes (for insulin, iron, ferritin, serum fatty acids profile, and serum chemistry), EDTA BD Vacutainer blood collection tubes (for erythrocyte fatty acid profile), and Lithium Heparin BD Vacutainer blood collection tubes (for plasma chemistry, including triglycerides). Blood tubes were centrifuged at 3000 rpm for 10 minutes within 30–60 minutes of collection and chilled during processing until shipment. The EDTA and lithium heparin tubes were shipped on cold packs to the reference laboratories. Remaining serum/plasma was transferred to cryovials and stored at -80°C until shipment on dry ice via overnight courier to the reference laboratories.

Serum and red blood cell membrane fatty acid profiles were performed by the Genetics Laboratories at the Kennedy Krieger Institute. Fatty acids were analyzed by capillary gas chromatography/mass spectrometry of pentaflourobenzyl bromide fatty acid esters using an AT-Silar-100 column (Grace, Columbia, Maryland 21044) as previously described [29]. For red blood cells only, the lipids were extracted with hexane:isopropanol before analysis. A summary of standards used and a year of quality control data, showing average coefficient of variation percent (CV%) from RBC and plasma controls, are provided as supporting information (S1 Table). Each run was required to pass clinical laboratory quality control before the data were released. CV% were typically under 10%. Percent fatty acids in serum was used as a sturdier index to help reduce potential variability in serum among study dolphins.

Iron, TIBC, and ferritin were analyzed at the Kansas State Veterinary Diagnostic Laboratory by colorimetric analysis on the Roche Cobas Mira (Roche Diagnostics, Indianapolis, Indiana 46250) per the manufacturer’s protocol. Plasma triglycerides and glucose were directly measured using the Roche Cobas 8000 system (Roche Diagnostics, Indianapolis, Indiana 46250) per the manufacturers’ protocol. Glucose was measured photometrically at the Animal Health Diagnostic Center at Cornell University on the Roche Diagnostics Modular Analytics P Module clinical chemistry analyzer (Roche Diagnostics, Indianapolis, IN 46250). Total insulin was analyzed at ARUP Laboratories by ultrafiltration/quantitative chemiluminescent immunoassay on the Siemens ADVIA Centaur Immunoassay system (Siemens Medical Solutions USA, Inc., Malvern, Pennsylvania 19355).

### Identification of potential fish-based dietary fatty acids associated with insulin

Statistical analyses were conducted using World Programming System software (World Programming Ltd., Hampshire, United Kingdom). A general linear model was used to test for associations between insulin and 55 individual serum fatty acids. The 31 (56%) fatty acids that were associated with insulin were included in a multivariate, stepwise regression model to determine independent predictors of insulin. Among the six (11%) fatty acids that were independent predictors of insulin, a Wilcoxon rank-sum test was used to compare fatty acid levels between MMP and Sarasota Bay dolphins; three (5%) of the six fatty acid had lower levels in MMP compared to Sarasota Bay dolphins. To identify potentially low fish-based fatty acids that may be corrected through a modified diet, the term, ‘targeted dietary fatty acids’ for the remaining study was defined as fatty acids that were independent predictors of insulin and had significantly lower levels in MMP dolphins compared to Sarasota Bay dolphins. A summary of the statistical process and associated data used to identify this study’s targeted fatty acids are provided as Supporting Information (S2 Table). Significance was defined as a *P* value less than 0.05.

### Comparisons of targeted serum fatty acids and metabolic panel values by study group

Age, insulin, glucose, triglycerides, iron, transferrin saturation, ferritin, and targeted percent serum fatty acids (C17:0, C20:4n6, and C22:0) were compared between MMP and Sarasota Bay dolphins using Wilcoxon rank-sum tests. Because MMP dolphins were significantly older than Sarasota Bay dolphins, comparisons of glucose, triglycerides, iron, transferrin saturation, ferritin, and targeted percent serum fatty acids controlled for age by using an analysis of covariance with age as a covariate.

### Comparisons of fatty acid and other nutrient content among fish types and dairy products

Fish fatty acid profiles and iron measurements were performed by Covance Laboratories (Madison, Wisconsin 53703). Each of the following fish types was mixed with water and homogenized for uniformity: capelin from Canada and Iceland (*Mallotus villosus*), Atlantic croaker (*Micropogonias undulatus*), herring (*Clupea harengus*), mackerel (*Scomber japonicus*), pinfish (*Lagodon rhomboides*), squid (*Loligo opalescens*), and striped mullet (*Mugil cephalus*). The lipid was extracted, saponified with 0.5N methanolic sodium hydroxide, and methylated with 14% BF3-methanol. The resulting methyl esters of the fatty acids were extracted with heptane. An internal standard was added prior to the lipid extraction. The methyl esters of the fatty acids were analyzed by gas chromatography using external standards for quantitation. Iron was measured by ICP Emission Spectrometry according to the Official Methods of Analysis of AOAC INTERNATIONAL, 18th Ed., Method 984.27 and 985.01, AOAC INTERNATIONAL, Gaithersburg, Maryland, USA, (2005). (Modified) (Covance, Madison, Wisconsin 53703). Given the previously published presence of C17:0 in dairy products, this fatty acid was also measured using the same methodology outlined above for fish in samples of nonfat milk, nonfat yogurt, 2% fat milk, whole fat milk, whole fat yogurt, and butter.

### 24-week feeding study with MMP dolphins

Based upon the study’s results, resolution of potentially low C17:0, C20:4n6, and C22:0 was sought in MMP dolphins by feeding six dolphins (among the initial 30) a diet that reduced capelin intake and introduced either pinfish or mullet. The modified diet maintained the same total kilocalories of the baseline diet but changed the percent of total fish fed (bulk weight) to approximately 25% capelin, 25% pinfish or mullet, and a 50% combination of herring, mackerel, and squid. Comparisons of total daily fatty acid intake between baseline and study diets were conducted using a Wilcoxon rank-sum test. 2h postprandial blood sample collection and analyses were the same as those described above. To evaluate potential confounding effects of the environment outside of the feeding study, archived, routine monthly samples collected from four MMP reference dolphins that were housed in the same environment were assessed for glucose, triglycerides, ferritin, and percent serum fatty acids. Reference dolphins had routine, fasted blood samples collected during or near to weeks 0, 12, 18, and 24 of the feeding study. Insulin levels were not measured for the reference group since they were overnight fasted samples; previous studies have demonstrated that fasted insulin levels (versus 2h postprandial levels) in dolphins with or without metabolic syndrome near zero [10].

Changes in percent serum for the targeted fatty acids (C17:0, C20:4n6, and C22:0), as well as insulin, glucose, triglycerides, iron, transferrin saturation, and ferritin, were assessed among feeding study dolphins during weeks 3, 6, 12, 18 and 24 and compared to week 0 using pairwise comparison t-tests. Erythrocyte membrane fatty acids were measured during weeks 3, 6, 12, 18, and 24 (samples for this test were not collected during week 0); pairwise comparison t-tests compared erythrocyte membrane fatty acid levels at weeks 6 through 24 to week 3. Given the apparent tightening or normalization of glucose, triglycerides, and insulin values among feeding study dolphins by week 24, measures of spread (standard deviation, SD, and coefficient of variance, or CV) were compared between weeks 0 and 24 for glucose, triglycerides, and insulin; outcomes for glucose and triglycerides were compared to the reference dolphin group. CV was calculated as follows: *standard deviation ÷ mean*. Dolphin E, the male study dolphin that did not have decreasing insulin during the feeding study, was also experiencing rut (strong reproductive behavior) accompanied by high serum testosterone levels, which may have been a contributor to his sustained high insulin levels. Based upon decreasing high ferritin levels identified in dolphins on the modified diet (and to assess if downward trends were occurring previous to the study), archived routine serum samples from these six dolphins, representing four sampling periods throughout the year previous to and leading up to the study, were tested for ferritin and compared with week 0.

### Tested associations among ferritin, C17:0, and C20:4n6

Given the detected decrease in ferritin among dolphins on the modified diet, associations between serum and RBC (total and percent) C17:0 and C20:4n6 were tested with serum ferritin using a general linear model. This analysis was limited to Sarasota Bay dolphins since 18 of 19 dolphins in this group had all relevant measurements collected (the initial 30 MMP dolphins did not have RBC fatty acid levels measured). Additionally, Sarasota Bay dolphins had a more normal distribution of ferritin values compared to MMP dolphins. Due to their significant associations with ferritin, linear associations were then tested between total serum C17:0 and C20:4n6 with serum ferritin using simple linear regression. Since both total fatty acids had linear associations with ferritin, stepwise regression was used to assess total serum C17:0 and C20:4n6 as independent predictors of ferritin, controlling for age.

## Results

### Serum fatty acids and insulin

Of 55 fatty acids assessed (S2 Table), percent serum of the following thirty individual fatty acids were associated with insulin in dolphins: C10:0, C14:0, C15:0, prisantic acid, C16:1n9, C17:0, C17:1, C18:1n9, C18:1n7, C18:2n6, C20:0, C20:1, C20:3n9, C20:3n7, C20:2n6, C20:4n6, C21:0, C22:1n9, C23:0, C22:5n6, C22:4n6, C22:5n3, C24:0, C24:2, C25:0, C25:1, C26:0, C26:2, C28:0, C29:0. Of these, six fatty acids (C16:1n9, C17:0, C18:1n9, C20:2n6, C20:4n6, and C22:0) were independent predictors of insulin. Among these six, three (C17:0, C20:4n6, and C22:0) had lower levels in MMP compared to Sarasota Bay dolphins. As such, the remaining focus of this study was on the three following potential dietary and correctable targeted fatty acids: C17:0 (heptadecanoic acid or margaric acid), C20:4n6 (arachidonic acid), and C22:0 (behenic acid). A more detailed table of these results is provided (S2 Table).

Similar to what has been previously reported using a different study time period among the same populations [12], MMP dolphins were older than Sarasota Bay dolphins. Correcting for age, MMP dolphins had higher insulin, triglycerides, ferritin, iron, and transferrin saturation (Table 1). When comparing the three targeted fatty acids and controlling for age, MMP dolphins had lower mean C17:0, C20:4n6, and C22:0 compared to Sarasota Bay dolphins (Table 1).

**Table 1:** Comparisons of demographics, metabolic health indicators, and targeted serum fatty acids between Navy Marine Mammal Program and Sarasota Bay, Florida bottlenose dolphins. Comparisons of metabolic variables and targeted serum fatty acids controlled for age.

Demographic or blood-based variable	MMP (n = 30)	Sarasota Bay(n = 19)	*P* value
Age (years)	26 ± 12	13 ± 9	0.002
Sex (% females)	15 (50%)	12 (63%)	0.37
**Metabolic variable**			
Insulin (μIU/ml)	11 ± 12	2 ± 5	0.04
Serum glucose (mg/dl)	104 ± 15	117 ± 10	0.02
Triglycerides (mg/dl)	149 ± 59	78 ± 26	< 0.0001
Ferritin (ng/ml)	3,878 ± 3,754	219 ± 184	0.005
Iron (μg/dl)	177 ± 57	109 ± 48	0.0003
Transferrin saturation (%)	56 ± 20	33 ± 11	< 0.0001
**Targeted serum fatty acid (%)**			
C17:0	0.3 ± 0.1	1.3 ± 0.4	< 0.0001
C20:4n6	4.1 ± 1.0	17.4 ± 2.3	< 0.0001
C22:0	0.2 ± 0.04	0.7 ± 0.2	< 0.0001

### Targeted fatty acid and other nutrient content in fish and dairy products

Capelin, the primary fish type fed to MMP dolphins, had no detectable C17:0 (< 0.007 g/100g), while fish types that are commonly part of Sarasota Bay dolphin diets did have C17:0 (croaker = 39, pinfish = 41, and mullet = 67 mg/100g). Herring and mackerel fed to MMP dolphins as a relatively small part of their diet had detectable C17:0 (19 and 22 mg/100g, respectively). There was no detectable C17:0 in squid. C20:4n6 was present in fish at the following levels (mg/dl, highest to lowest): mullet (164), croaker (90), mackerel (83), pinfish (77), herring (74), squid (17), and capelin (9 to 14). C22:0 was not detectable in capelin, squid, mackerel, or mullet. It was present in other fish at the following levels (mg/dl, highest to lowest): pinfish (14), croaker (13), and herring (10). Tested ranges were comparable for all fish types for percent moisture (74–83%), protein (13–21%), fat (0.6–3.6%), and carbohydrates (0–1.2%), except herring, which had 67% moisture, and 14% fat. Herring intake was not altered substantially in the feeding study.

Due to the known presence of C17:0 in dairy products, C17:0 levels were measured in off-the-shelf dairy products. The amount of C17:0, from highest to lowest (mg/100g), was butter (423), whole fat yogurt (31), whole fat milk (19), and 2% fat milk (10). C17:0 was not detected in either nonfat milk or nonfat yogurt. C20:4n6 was not detected in nonfat milk, nonfat yogurt, and whole yogurt; it was present in 2% milk, whole milk, and butter at levels of 3, 6, and 135 mg/100g. Behenic acid was not detected in the tested dairy products.

### 24-week feeding study

Based on the results above, the diets of six MMP dolphins were modified to increase percent serum of the targeted fatty acids by decreasing capelin and introducing pinfish and mullet to their diet while maintaining the same kilocalories. Comparisons of daily fatty acid intake of the dolphins’ original and modified diets are provided (Table 2), including demonstrated increased intake of C17:0 from a daily mean of 400 to 1,700 mg (greater than a four-fold increase) and C20:4n6 from a daily mean of 2,000 to 5,000 mg with the modified diet. There was no significant change in C22:0 intake with the modified diet.

**Table 2:** Comparisons of dietary fatty acid (g) intake between original and modified diets for six bottlenose dolphins.

Fish-based nutrient	Original diet–total daily intake	Modified diet—total daily intake	*P* value
**Targeted fatty acids (g)**			
Heptadecanoic acid (C17:0)	0.4 ± 0.2	1.7 ± 0.5	0.006
Arachidonic acid (20:4n6)	2 ± 1	5 ± 2	0.006
Behenic acid (C22:0)	0.2 ± 0.1	0.3 ± 0.3	0.43
**Other fatty acids (g)**			
Myristic acid (C14:0)	22 ± 6	18 ± 5	0.23
Pentadecanoic acid (C15:0)	1 ± 0.4	5 ± 4	0.007
Palmitic acid (C16:0)	62 ± 22	67 ± 22	0.71
Stearic acid (C18:0)	8 ± 3	12 ± 3	0.03
Oleic acid (C18:1n9)	55 ± 25	50 ± 30	0.79
Linoleic acid (C18:2)	7 ± 2	6 ± 2	0.37
Linolenic acid (C18:3)	0.8 ± 0.5	1.5 ± 0.4	0.04
Gamma-linolenic (C18:3n3)	0.2 ± 0.1	0.6 ± 0.5	0.16
Arachidic acid (C20:0)	0.3 ± 0.2	0.7 ± 0.4	0.32
Eicosadienoic acid (C20:2n6)	0.3 ± 0.1	0.5 ± 0.2	0.14
Eicosapentaenoic acid (20:5n3)	40 ± 13	35 ± 11	0.43
Erucic acid (C22:1n9)	6 ± 1	3 ± 1	0.006
Docosapentaenoic acid (C22:5n6)	4 ± 1	6 ± 1	0.009
Docosahexaenoic acid (C22:6n3)	38 ± 10	42 ± 11	0.63
Lignoceric acid (C23:0)	0	0.2 ± 0.1	0.005
Omega 3 fatty acids	86 ± 25	89 ± 22	0.79
Omega 6 fatty acids	9 ± 3	12 ± 2	0.11
Omega 6:3 fatty acids	0.1 ± 0.01	0.1 ± 0.03	0.006
Omega 9 fatty acids	101 ± 33	72 ± 37	0.23
Total cis-unsaturated fatty acids	143 ± 99	207 ± 66	0.14
Total trans-unsaturated fatty acids	10 ± 3	8 ± 2	0.16
Monosaturated fatty acids	143 ± 46	111 ± 45	0.27
Polyunsaturated fatty acids	92 ± 27	96 ± 22	0.56

**Fig 1:**
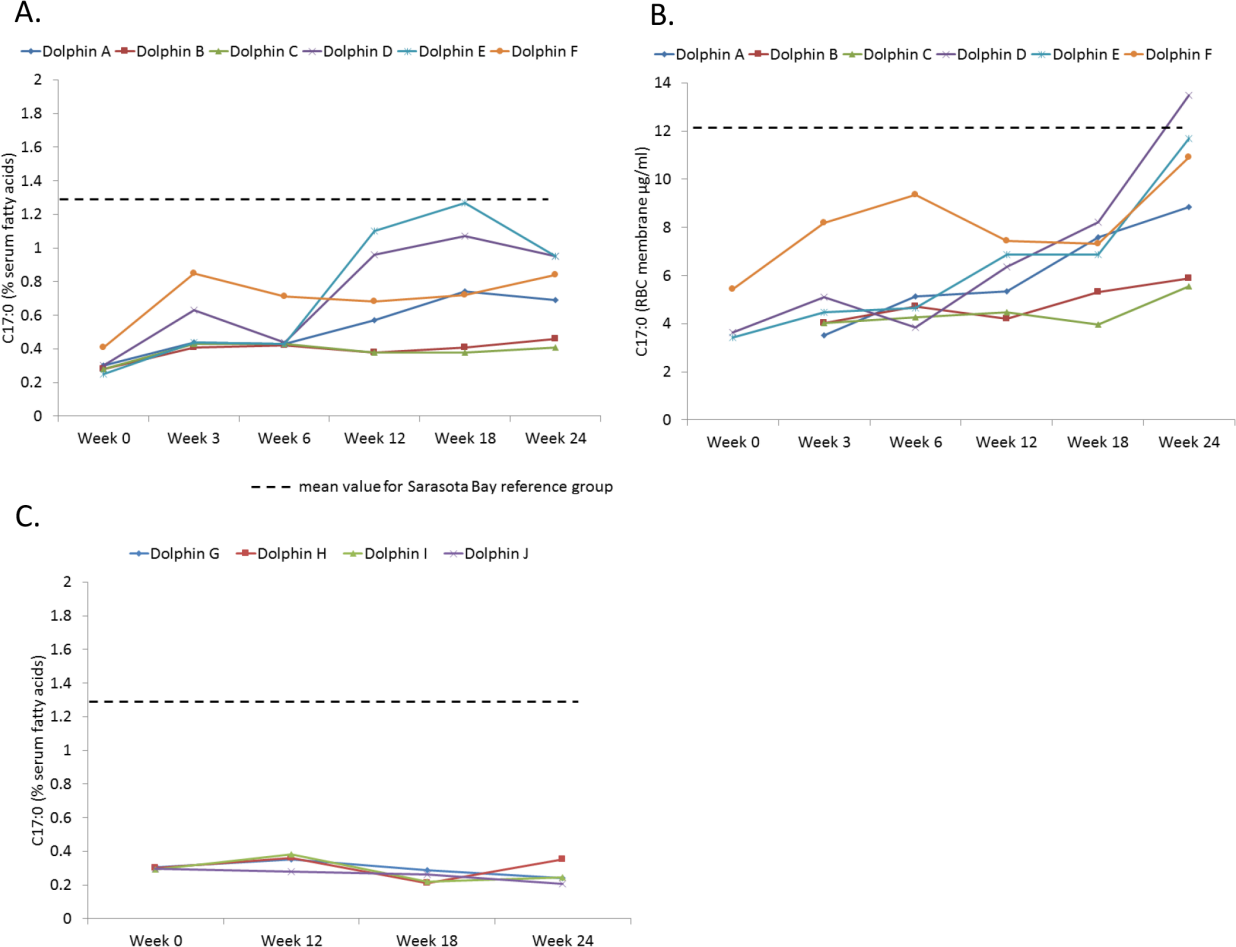
Heptadecanoic acid (C17:0) levels in bottlenose dolphins during a 24-week feeding study, including A) percent serum C17:0 of dolphins on a modified diet with an average increased dietary C17:0 intake from 400 to 1,700 mg/day (n = 6), B) total erythrocyte membrane C17:0 levels of dolphins on the modified C17:0 diet (n = 6), and C) percent serum C17:0 of reference dolphins not on modified diet (n = 6). The dashed line indicates the mean value for the Sarasota Bay reference group.

Percent serum fatty acid levels of C17:0, C20:4n6 and C22:0 for dolphins on the modified diet were higher during weeks 3, 6, 12, 18, and 24 compared to week 0 (Table 3). While percent serum C17:0 increased above baseline by week 3, significant increases in erythrocyte membrane levels did not occur until week 24, potentially indicating the duration of time it took to integrate higher dietary fatty acid levels into the dolphin’s red blood cell membranes (Fig 1A and 1B). While erythrocyte membrane C17:0 levels were 12 ± 3 μg/ml in the Sarasota Bay reference population, feeding study dolphin levels were as follows: 5 ± 2 μg/ml (week 3), 5 ± 2 μg/ml (week 6), 6 ± 1 μg/ml (week 12), 7 ± 2 μg/ml (week 18), and 9 ± 3 μg/ml (week 24). There was no change in percent serum C17:0, C20:4n6 and C22:0 among reference dolphins during the study (Table 4).

**Table 3:** Blood-based indicators of metabolic syndrome and fatty acid values during 24-week feeding study with six bottlenose dolphins on modified diet and comparisons of values during weeks 3, 6, 12, 18, and 24 compared to baseline week 0. Significant *P* values are provided.

Blood variable	Wild reference dolphins	Week 0	Week 3	Week 6	Week 12	Week 18	Week 24
**Serum fatty acids (%)**							
Heptadecanoic acid (C17:0)	1.3 ± 0.4	0.3 ± 0.1	0.5 ± 0.2	0.5 ± 0.1	0.7 ± 0.3	0.8 ± 0.4	0.7 ± 0.2
			*P* = 0.007	*P* = 0.001	*P* = 0.03	*P* = 0.03	*P* = 0.007
Arachidonic acid (20:4n6)	17 ± 2	4 ± 1	6 ± 2	7 ± 2	10 ± 4	10 ± 3	10 ± 3
			*P* = 0.005	*P* = 0.001	*P* = 0.01	*P* = 0.005	*P* = 0.004
Behenic acid (C22:0)	0.7 ± 0.2	0.15 ± 0.04	0.21 ± 0.06	0.25 ± 0.07	0.28 ± 0.06	0.26 ± 0.04	0.29 ± 0.04
			*P* = 0.004	*P* = 0.0008	*P* = 0.003	*P* = 0.002	*P* < 0.0001
**Metabolic health indicators**							
Insulin (μIU/ml)	2 ± 5	24 ± 21	17 ± 7	19 ± 23	22 ± 25	20 ± 21	16 ± 20
Insulin (μIU/ml)^1^	2 ± 5	19 ± 18	14 ± 5	10 ± 5	12 ± 9	11 ± 5	8 ± 2
Plasma glucose (mg/dl)	102 ± 15	109 ± 21	103 ± 13	110 ± 14	109 ± 17	97 ± 12	95 ± 6
Triglycerides (mg/dl)	78 ± 26	132 ± 81	166 ± 67	112 ± 37	119 ± 30	117 ± 45	97 ± 28
Iron (μg/dl)	109 ± 48	162 ± 64	153 ± 35	152 ± 52	160 ± 77	153 ± 31	177 ± 48
Iron (μg/dl) ^2^	109 ± 48	132 ± 23	131 ± 4	127 ± 11	114 ± 38	136 ± 22	153 ± 29
Ferritin (ng/ml)	219 ± 184	3697 ± 6813	4235 ± 8198	2954 ± 5271	1160 ± 1905	1218 ± 1695	2201 ± 4656
Ferritin (ng/ml)^2^	219 ± 184	373 ± 52	341 ± 48	323 ± 52	263 ± 40	250 ± 67	243 ± 58
			*P* = 0.0009		*P* = 0.02	*P* = 0.005	*P* = 0.002
Transferrin saturation (%)	33 ± 11	50 ± 25	49 ± 17	50 ± 26	52 ± 33	51 ± 19	60 ± 22
Transferrin saturation (%)^2^	33 ± 11	39 ± 5	40 ± 8	38 ± 8	31 ± 9	40 ± 7	49 ± 14
Ceruloplasmin (mg/dl)	18 ± 6^3^	19 ± 5	18 ± 5	19 ± 4	23 ± 8	20 ± 5	19 ± 5
Haptoglobin (mg/dl)	17 ± 6^3^	11 ± 3	12 ± 5	12 ± 3	14 ± 6	14 ± 6	9 ± 9

^1^Results when removing dolphin with high testosterone and breeding behavior during study.

^2^Two outlier high ferritin dolphins, which also had decreasing ferritin during the feeding study, were removed to enable comparisons of mean values during the study (see text results).

^3^Based upon previously reported results on wild, free-ranging dolphins in the Indian River Lagoon (Mazzaro et al. 2012).

**Table 4:** Targeted percent serum fatty acids and blood-based metabolic health indices in reference dolphins (n = 4) during 24-week feeding study, comparing values from weeks 12, 18 and 24 to baseline week 0. No values from weeks 12, 18, and 24 were significantly different than week 0.

Blood variable	Week 0	Week 12	Week 18	Week 24
Heptadecanoic acid (C17:0)	0.30 ± 0.05	0.31 ± 0.07	0.29 ± 0.07	0.26 ± 0.04
Arachidonic acid (20:4n6)	5.2 ± 0.4	5.5 ± 0.8	6.0 ± 0.5	5.3 ± 0.7
Behenic acid (C22:0)	0.2 ± 0.02	0.2 ± 0.01	0.2 ± 0.04	0.2 ± 0.05
Glucose (mg/dl)	103 ± 12	102 ± 28	108 ± 33	99 ± 14
Triglycerides (mg/dl)	49 ± 20	57 ± 11	55 ± 15	91 ± 98
Ferritin (ng/ml)	503 ± 107	415 ± 93	384 ± 50	409 ± 47

**Fig 2:**
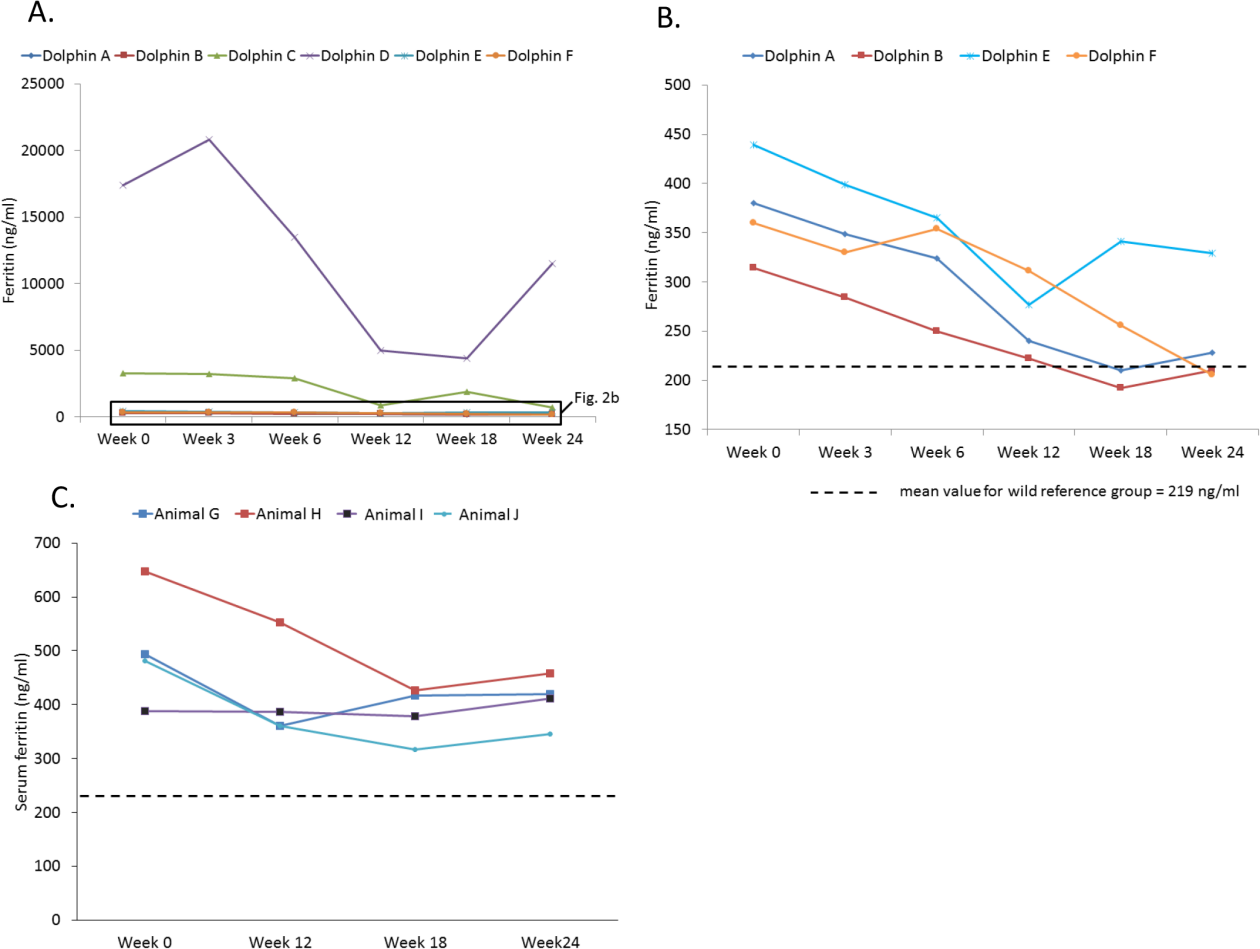
Serum ferritin levels in bottlenose dolphins during a 24-week feeding study, including A) dolphins on a modified diet with an average increased dietary intake of heptadecanoic acid (C17:0) from 400 to 1,700 mg/day (n = 6), B) dolphins on the modified diet (removing two high outlier dolphins), and C) reference dolphins. The dashed line indicates the mean value for the Sarasota Bay reference group.

Serum ferritin levels decreased in all six dolphins on the modified diet, with weeks 3 through 24 having lower levels than week 0 (Table 3). When removing the two feeding study dolphins which had extremely high ferritin (levels in the upper thousands to tens of thousands *versus* hundreds), which limited statistical interpretations for the study group, week 24 had the lowest mean serum ferritin (243 ± 58 ng/ml), nearing Sarasota Bay dolphins’ mean value of 219 ± 184 ng/ml (Fig 2). The highest ferritin dolphins decreased their ferritin levels (week 0 to week 24) from 3,289 to 726 ng/ml (Animal C) and 17,400 to 11,512 ng/ml (Animal D). Among feeding study dolphins, there was no significant change in ferritin during the year before the study compared to week 0, as well as among the subset of the four non-outlier ferritin dolphins (mean ferritin for these four dolphins 10 to 12 months before the study = 350 ± 39 ng/ml, 7 to 9 months pre-study = 371 ± 133 ng/ml, 4 to 6 months pre-study = 394 ± 177 ng/ml, and 1 to 3 months pre-study = 546 ± 157 ng/ml; *P* value = 0.25, 0.96, 0.77 and 0.10, respectively), demonstrating that downward trends were not occurring in cases before the feeding study. Due to the decrease in serum ferritin in all six feeding study dolphins, indices of acute inflammation (ceruloplasmin and haptoglobin) were assessed. Despite decreases in ferritin, there were no differences in these two proteins during any of the study weeks compared to week 0, supporting that decreased ferritin was likely not due to changes in acute inflammation (Table 1). Among the reference dolphins, no changes were found in targeted fatty acids, ferritin, insulin, glucose, or triglycerides when comparing weeks 12, 18, and 24 to week 0 (Table 4). Among fish types fed, squid and capelin had the lowest iron levels (less than 3 and 10 ppm, respectively), and pinfish and mullet had the highest iron levels (34 and 40 ppm, respectively), demonstrating that decreased dietary iron intake was not the cause of decreasing ferritin.

**Fig 3:**
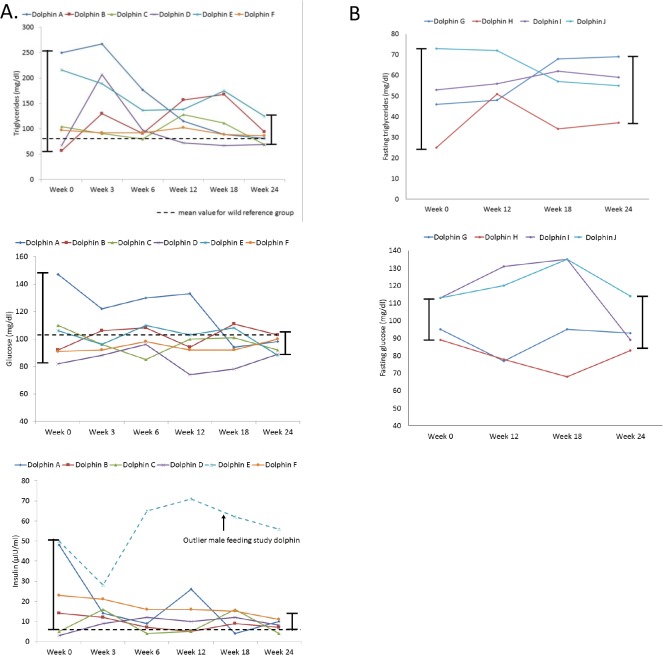
Blood-based indicators of metabolic syndrome and measures of spread in bottlenose dolphins during a 24-week feeding study, including A) triglycerides, glucose, and insulin (for insulin, decrease in spread is only present when excluding one outlier dolphin), and B) triglycerides and glucose in reference dolphins. The dashed line indicates the mean value for the Sarasota Bay reference group.

Dolphins fed the modified diet had a decrease in measures of spread (i.e. normalization) for triglycerides, glucose, and insulin that trended consistently from weeks 0 to 24 (Table 3, Fig 3). The standard deviation for glucose and triglycerides decreased from 23 to 6 mg/dl and 81 to 21 mg/dl, respectively. In comparison, reference dolphins had an increase in standard deviation from weeks 0 to 24 (glucose increased from 12 to 14, and triglycerides increased from 20 to 98) (Table 4). The coefficient of variation (C.V.) from week 0 to week 24 for dolphins on the modified diet decreased from 22% to 6% for glucose and 61% to 24% for triglycerides. When limiting to five study dolphins (excluding the outlier sixth male dolphin that maintained high insulin possibly due to rut behavior and associated high testosterone throughout the study), the insulin standard deviation for dolphins on the modified diet decreased dramatically from 18 to 3 μIU/ml. When limiting to five study dolphins (excluding the sixth dolphin that was experiencing rut behavior), the insulin C.V. decreased from 100% to 38%. The decrease in measures of spread for these three key variables are visually apparent in Fig 3.

### Tested associations among ferritin, C17:0, and C20:4n6

**Fig 4:**
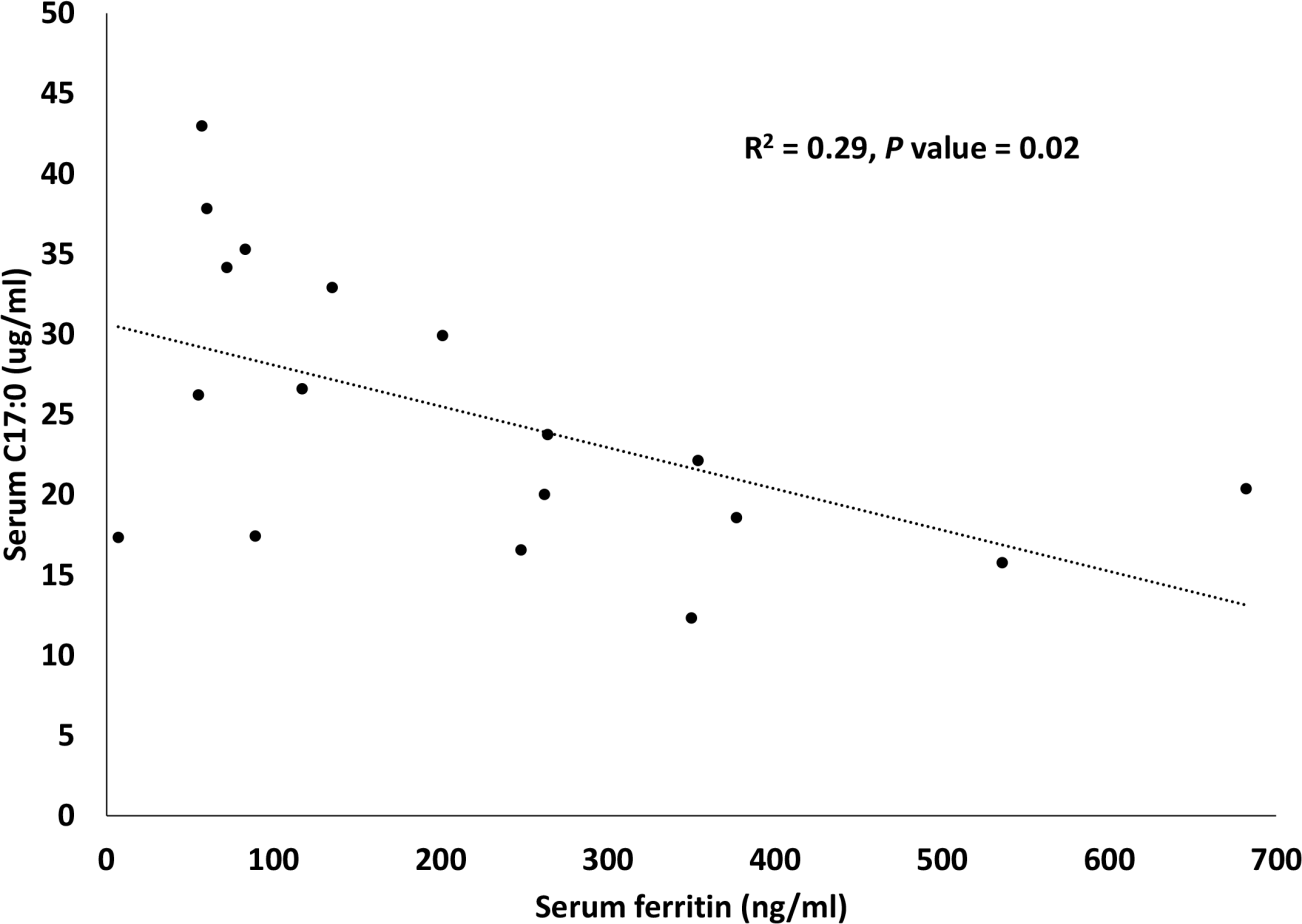
Inverse linear association between total serum C17:0 and serum ferritin in Sarasota Bay dolphins (n = 19).

A summary of tested associations between serum and RBC (total and percent) C17:0 and C20:4n6 with ferritin is provided in Table 5. Total serum C17:0 (*P* = 0.02) and total serum C20:4n6 (*P* = 0.03) were associated with serum ferritin. Total serum C17:0 (R^2^ = 0.29, *P* = 0.02) (Fig 4) and total serum C20:4n6 (R^2^ = 0.26, *P* = 0.03) had inverse, linear relationships with ferritin. Stepwise regression, including age as a covariate, demonstrated that only total serum C17:0 was an independent predictor of serum ferritin in dolphins (*P* = 0.02).

**Table 5:** Tested linear associations between C17:0 and C20:4n6 with ferritin in Sarasota Bay dolphins (n = 19) using a general linear model.

Fatty acid	Association with serum ferritin (*P* value)
Percent serum C17:0	0.22
Total serum C17:0	0.02
Percent RBC membrane C17:0	0.14
Total RBC membrane C17:0	0.27
Percent serum C20:4n6	0.09
Total serum C20:4n6	0.03
Percent RBC membrane C20:4n6	0.16
Total RBC membrane C20:4n6	0.11

## Discussion

Our study identified low blood levels of a saturated fatty acid, C17:0, which when corrected by a modified diet, was associated with decreasing high ferritin and normalization of glucose, triglycerides, and insulin. Importantly, when dolphins with high ferritin had increased C17:0 dietary intake by changing fish types fed, ferritin decreased by week 3, and glucose, triglycerides, and insulin normalized by week 24. Interestingly, higher C17:0 was confirmed an independent predictor of lower ferritin in the wild reference population. Since high ferritin is associated with metabolic syndrome in both dolphins and humans, and resolution of high ferritin with phlebotomy in humans improves insulin resistance, this study introduces a novel hypothesis that low dietary and serum C17:0 may be associated with high ferritin and subsequent metabolic syndrome [12,30,31].

C17:0, also called margaric or heptadecanoic acid, is a saturated fatty acid present in bovine milk fat and was the original component of margarine (hence, margarine’s name) in the late 1800s [32,33]. C17:0 in margarine, however, was replaced with less costly and more readily available plant-based and trans-fatty acids [32]. When off the shelf dairy products were tested in the current study, C17:0 was highest in butter and whole fat yogurt and not detectable in nonfat dairy products. Butter had 10-fold higher levels of C17:0 compared to the next highest C17:0 foods. Interestingly, despite widespread recommendations for consumers to avoid high fat foods (including whole fat dairy products), previous studies in humans have demonstrated that whole fat dairy consumption is associated with multiple health benefits, including lower risks of insulin resistance, metabolic syndrome, and type 2 diabetes [34–38]. To date, the mechanism of the benefits of dairy products on human metabolism has not been fully determined [39]. Our study findings suggest that C17:0 may be a key player in the metabolic benefits of dairy and other dietary products containing C17:0.

This study’s finding of potential metabolic benefits of C17:0 is supported in the human literature. Higher erythrocyte membrane or plasma phospholipid C17:0 levels, among other identified fatty acids, have been identified as biomarkers of or potential protective factors against development of metabolic syndrome, type 2 diabetes, and associated inflammation in humans [40–44]. C17:0 is considered a fatty acid acquired primarily through the diet, and people can successfully raise their plasma C17:0 levels by increasing their dietary intake of C17:0 through dairy products [43,45,46]. In fact, C17:0 measurements in blood are used as a successful short-term marker for how much dairy products human study subjects ingest [43,45,46].

C17:0, although most commonly reported in bovine milk fat, is also found in a variety of fish [47]. As demonstrated in the current study, C17:0 levels vary among fish, from no detectable levels in this study’s capelin to 2.3% of total lipids in shrimp [47–49]. C17:0 content can be higher in the same fish species during the summer versus winter; and among freshwater versus seawater fish species [47,49]. Given our study’s discovery of improved ferritin and normalizing glucose, triglycerides, and insulin based upon the type of fish fed, and inconsistencies of previous human studies related to the metabolic benefits and risks of dietary fish, we propose that these health differences may lie in the differences of fatty acid content in dietary fish, including C17:0 [3,4].

In addition to C17:0, low levels of two additional fatty acids may be important biomarkers for elevated insulin in dolphins. In this study, higher percent serum arachidonic acid (C20:4n6), an omega-6 polyunsaturated fatty acid and behenic acid (C22:0), another saturated fatty acid, were associated with lower insulin. In humans, arachidonic acid supplementation can prevent alloxan-induced diabetes, but other potential benefits to metabolism has not been clear [50]. Although total serum C20:4n6 level was associated with ferritin in this study, C20:4n6 fell out as an independent predictor of ferritin, and C17:0 remained. Better understanding how these and other dietary fatty acids, as well as other nutrients, may benefit metabolism can be gained by larger scale population modified diet studies with dolphins.

There are several important limitations to the current study. First, study dolphins from MMP and Sarasota Bay live in the open ocean, and confirmed dietary intake was limited to fish fed to MMP dolphins. MMP dolphins live in netted enclosures in San Diego Bay, and changing populations of local fish are readily available prey. While MMP dolphins can eat local fish, observation of feeding behaviors by MMP’s animal care staff support that the majority of dietary fish are those that are fed by the staff. Further, reference dolphins in the same population and environment did not demonstrate the same decreases in serum ferritin and normalization of glucose and triglycerides, supporting that this study’s findings were due to the diet change and not the environment. Second, the potential impact (or cumulative impacts) of other nutrients in the modified diet on the study dolphins can not be ruled out. Third, while an association was found between increasing C17:0 and improved blood-based metabolic profiles, this study demonstrated an association versus a definitive cause of improved metabolism. Finally, while increased dietary intake of C17:0 in the modified diet was associated with increased blood levels of C17:0, supporting that this increase was due to dietary intake, there is some evidence in other species that C17:0 may also be produced endogenously. Future studies using concentrated C17:0 in a larger population may help address this study’s limitations.

In conclusion, this study with dolphins is the first to propose low blood and dietary C17:0, a saturated fatty acid, as a means to detect elevated insulin with associated high ferritin. Further, a modified diet with increased daily C17:0 intake in a small number of dolphins was associated with resolving metabolic syndrome and decreasing ferritin. This is the first report of lower total serum C17:0 as an independent predictor of higher ferritin. Future research with human populations is needed to assess relationships among dietary intake of foods high in C17:0 and potential beneficial impacts of this saturated fat on metabolic syndrome, hyperferritinemia, and diabetes. Importantly, the potential benefits of increased dietary C17:0 intake need to be measured carefully against total caloric intake and increased intake of other fats which may not be beneficial.

## Supporting Information

S1 Table Summary of Quality Control and Standards Used for Fatty Acid Measurements. Data includes quality control results over a 12-month period at the reference laboratory.
(XLSX)

S2 Table Summary of Process to Identify Targeted Fatty Acids. The stepwise statistical process to identify the targeted fatty acids from the 55 fatty acid profile in bottlenose dolphins (*Tursiops truncatus*) was as follows: 1) associations between individual fatty acids and insulin using a general linear model, 2) independent predictors of insulin using multivariate stepwise regression, 3) differences between Navy Marine Mammal Program and Sarasota Bay dolphins using an analysis of covariance controlling for age, and 4) serum percent levels lower in Navy Marine Mammal Program compared to Sarasota Bay dolphins.
(DOCX)
