# Supplementary material for: Increased Dietary Intake of Saturated Fatty Acid Heptadecanoic Acid (C17:0) Associated with Decreasing Ferritin and Alleviated Metabolic Syndrome in Dolphins
Source: PLoS One. 2015 Jul 22;10(7):e0132117. doi: 10.1371/journal.pone.0132117 (PMC4511797; doi:10.1371/journal.pone.0132117)
Supplement: S2 Table — The stepwise statistical process to identify the targeted fatty acids from the 55 fatty acid profile in bottlenose dolphins (Tursiops truncatus) was as follows: 1) associations between individual fatty acids and insulin using a general linear model, 2) independent predictors of insulin using multivariate stepwise regression, 3) differences between Navy Marine Mammal Program and Sarasota Bay dolphins using an analysis of covariance controlling for age, and 4) serum percent levels lower in Navy Marine Mammal Program compared to Sarasota Bay dolphins. (DOCX) [file pone.0132117.s002.docx]

| **Fatty acid** | **A. Association between fatty acid level and insulin (PROC GLM; model insulin = fatty acid)** | **B. Independent fatty acid predictors of insulin (PROC STEPWISE; model insulin = fatty acids / stepwise) Model with significant fatty acids = F Value = 12.6, P < 0.0001** | **C. Differences by study group, controlling for age**  **(PROC GLM; class group; model insulin = age group; means group)** | **D. Higher or lower mean values in Group A (MMP) versus Group B (Sarasota Bay)** |
| --- | --- | --- | --- | --- |
| C17:0 | Yes (*P* = 0.01) | Yes (*P* = .0005) | Yes (0.29 ± 0.06 and 1.3 ± 0.35) (*P* < 0.0001) | Lower |
| C20:4n6 | Yes (*P* = 0.002) | Yes (*P* = .002) | Yes (4.1 ± 1.0 and 17.4 ± 2.3) (*P* < 0.0001) | Lower |
| C22:0 | Yes (*P* = 0.0009) | Yes (*P* = 0.03) | Yes (0.18 ± 0.04 and 0.65 ± 0.15) (*P* < 0.0001) | Lower |
| C18:1n9 | Yes (*P* = 0.001) | Yes (*P* < .0001) | Yes (21 ± 1 and 17 ± 2) (*P* < 0.0001) | Higher |
| C20:2n6 | Yes (*P* = < 0.0001) | Yes (*P* = 0.03) | Yes (0.12 ± 0.02 and 0.09 ± 0.02) (*P* < 0.0001) | Higher |
| C16:1n9 | Yes (*P* = 0.0007) | Yes (*P* = .0002) | No |  |
| C10:0 | Yes (*P* = 0.04) | No |  |  |
| C14:0 | Yes (*P* = 0.002) | No |  |  |
| C15:0 | Yes (*P* = 0.02) | No |  |  |
| Prisantic | Yes (*P* = 0.04) | No |  |  |
| C17:1 | Yes (*P* = 0.01) | No |  |  |
| C18:1n7 | Yes (*P* = 0.0002) | No |  |  |
| C18:2n6 | Yes (*P* < 0.0001) | No |  |  |
| C20:0 | Yes (*P* = 0.0002) | No |  |  |
| C20:1n0 | Yes (*P* = 0.0003) | No |  |  |
| C20:3n9 | Yes (*P* = 0.005) | No |  |  |
| C20:3n7 | Yes (*P* = 0.003) | No |  |  |
| C21:0 | Yes (*P* = 0.0009) | No |  |  |
| C22:1n9 | Yes (*P* = 0.0005) | No |  |  |
| C23:0 | Yes (*P* = 0.002) | No |  |  |
| C22:5n6 | Yes (*P* = 0.002) | No |  |  |
| C22:4n6 | Yes (*P* = 0.005) | No |  |  |
| C22:5n3 | Yes (*P* = 0.04) | No |  |  |
| C24:0 | Yes (*P* = 0.001) | No |  |  |
| C24:2 | Yes (*P* = 0.004) | No |  |  |
| C25:0 | Yes (*P* = 0.002) | No |  |  |
| C25:1 | Yes (*P* = 0.002) | No |  |  |
| C26:0 | Yes (*P* = 0.0008) | No |  |  |
| C26:2 | Yes (*P* = 0.003) | No |  |  |
| C28:0 | Yes (*P* = 0.004) | No |  |  |
| C29:0 | Yes (*P* = 0.02) | No |  |  |
| C10:1 | No |  |  |  |
| C12:0 | No |  |  |  |
| C12:1 | No |  |  |  |
| C13:0 | No |  |  |  |
| C13:1 | No |  |  |  |
| C14:1 | No |  |  |  |
| C14:2 | No |  |  |  |
| C16:2 | No |  |  |  |
| C16:0 | No |  |  |  |
| C16:1n7 | No |  |  |  |
| Phytanic | No |  |  |  |
| C18:0 | No |  |  |  |
| C18:1n5 | No |  |  |  |
| C19:2n6 Conj | No |  |  |  |
| C18:3n6 | No |  |  |  |
| C18:0n3 | No |  |  |  |
| C20:3n6 | No |  |  |  |
| C20:5n3 | No |  |  |  |
| C22:2n6 | No |  |  |  |
| C22:6n3 | No |  |  |  |
| C24:1n9 | No |  |  |  |
| C24:1n9 | No |  |  |  |
| C26:1 | No |  |  |  |
| C:30 | No |  |  |  |
